# GMZ2 Vaccine-Induced Antibody Responses, Naturally Acquired Immunity and the Incidence of Malaria in Burkinabe Children

**DOI:** 10.3389/fimmu.2022.899223

**Published:** 2022-06-02

**Authors:** Sylvester Dassah, Bright Adu, Régis W. Tiendrebeogo, Susheel K. Singh, Fareed K. N. Arthur, Sodiomon B. Sirima, Michael Theisen

**Affiliations:** ^1^ Navrongo Health Research Centre, Navrongo, Ghana; ^2^ Department of Biochemistry and Biotechnology, Kwame Nkrumah University of Science and Technology, Kumasi, Ghana; ^3^ Department of Immunology, Noguchi Memorial Institute for Medical Research, University of Ghana, Legon, Ghana; ^4^ Department for Congenital Disorders, Statens Serum Institut, Copenhagen, Denmark; ^5^ Centre for Medical Parasitology at Department of Immunology, and Microbiology, University of Copenhagen, Copenhagen, Denmark; ^6^ Groupe de Recherche Action en Senté (GRAS), Ouagadougou, Burkina Faso

**Keywords:** GMZ2, MSP3-K1, GLURP-R2, *Plasmodium falciparum*, malaria vaccine, naturally acquired immunity

## Abstract

GMZ2 is a malaria vaccine candidate evaluated in a phase 2b multi-centre trial. Here we assessed antibody responses and the association of naturally acquired immunity with incidence of malaria in one of the trial sites, Banfora in Burkina Faso. The analysis included 453 (GMZ2 = 230, rabies = 223) children aged 12-60 months old. Children were followed-up for clinical malaria episodes for 12 months after final vaccine administration. Antibody levels against GMZ2 and eleven non-GMZ2 antigens were measured on days 0 and 84 (one month after final vaccine dose). Vaccine efficacy (VE) differed by age group (interaction, (12-35 months compared to 36-60 months), p = 0.0615). During the twelve months of follow-up, VE was 1% (95% confidence interval [CI] -17%, 17%) and 23% ([CI] 3%, 40%) in the 12 - 35 and 36 – 60 months old children, respectively. In the GMZ2 group, day 84 anti-GMZ2 IgG levels were associated with reduced incidence of febrile malaria during the follow up periods of 1-6 months (hazard ratio (HR) = 0.87, 95%CI = (0.77, 0.98)) and 7-12 months (HR = 0.84, 95%CI = (0.71, 0.98)) in the 36-60 months old but not in 12-35 months old children. Multivariate analysis involving day 84 IgG levels to eleven non-vaccine antigens, identified MSP3-K1 and GLURP-R2 to be associated with reduced incidence of malaria during the 12 months of follow up. The inclusion of these antigens might improve GMZ2 vaccine efficacy.

## Introduction

GMZ2 is a *Plasmodium falciparum* candidate vaccine, and is designed with the aim to emulate naturally acquired anti-malarial immunity (1). It is composed of conserved domains of two asexual blood-stage antigens of *P. falciparum*, glutamate-rich protein (GLURP) and merozoite surface protein (MSP) 3 which are major epitopes for antibodies (2, 3). The rationale for including these antigens is based on a series of immune epidemiological studies from diverse malaria endemic regions. Individuals living in malaria endemic areas gradually acquire immunity to clinical malaria (4, 5). This naturally acquired immunity (NAI) takes years of exposure to develop and is characterized by a low grade parasitemia in the presence of strong *P. falciparum*-specific immune responses (6). Immunoglobulin (Ig) G antibodies are thought to play a particularly important role in NAI (4, 7). Immuno-epidemiological studies of responses to GLURP and MSP3 have consistently demonstrated that high levels of specific antibodies are associated with protection against febrile malaria (8–16), in areas with different transmission intensity [ranging from >200 infective bites per person per year in a study in Senegal (16) to approximately 2 to 3 infective bites per person per year in a study in Sudan (15)], and with respect to their geographical locations, suggesting that GMZ2 might potentially confer immunity against clinical malaria in diverse endemic settings. Further, one of these studies suggested that antibodies against GLURP and MSP3 act in a complementary manner to control parasite multiplication (12). It is now generally accepted that protective immunity depends on a robust antibody response against multiple antigens (17–20), and it has been proposed that the magnitude and breadth of specific responses are critical in this respect (17).

While the exact immune mechanism(s) involved in NAI remains elusive, we and others have shown that monocyte mediated opsonic phagocytosis (OP) of *P. falciparum* blood-stage merozoites (8, 21, 22) and antibody-dependent cellular inhibition (ADCI) (23) are elicited during the acquisition of NAI. Recently, we further demonstrated that neutrophils may also help to eliminate circulating merozoites from blood during NAI (24).

Collectively, immuno-epidemiological studies together with pre-clinical studies in rodents and New World monkeys (25–27) led to the manufacturing and clinical testing of GMZ2 adjuvanted with alhydrogel^®^ (alum). GMZ2/alum was well tolerated and immunogenic in three phase 1 studies (28–30) and a phase 2b multi-centre trial in African children 12-60 months old (31). Overall, the trial showed that GMZ2 had a modest efficacy in the target population (31). In a sub-analysis we found that VE was higher in children 3–4 years of age (20% (4%, 33%)] compared to children 1–2 years of age [6% (-8%, 18%). An interaction with age is consistent with the proposed mode of action of GMZ2, which aims to mimic, boost, and broaden the breadth of NAI.

Here, we present the detailed immunological evaluation of samples from the GMZ2/alum phase 2b study collected at the Banfora site in Burkina Faso. Antibodies against GMZ2 and established targets of NAI were measured and evaluated against the incidence of clinical malaria.

## Methods

### Ethics Statement

Data for this study was obtained from the GMZ2/alum phase 2b clinical trial. The trial was monitored by the GMZ2 Scientific Coordinating Committee, local safety monitors, independent clinical monitors and an independent data safety monitoring committee (IDMC). The local Ethics Committees and regulatory authorities for each site and country approved the clinical trial protocol before the start of the trial. Signed informed consent was obtained from parent/guardian of children before their inclusion in the study. The protocol was registered with the Pan African clinical trial registry with registration number ATMR2010060002033537.

### Study Site and Design

The study used 453 (GMZ2 = 230, rabies=223) children’s specimen collected from Banfora, Burkina Faso in the GMZ2/alum phase 2b clinical trial. Malaria is endemic in Burkina Faso and occurs throughout the year, with seasonal peak between June and October, a period when rainfall is highest. *P. falciparum* is responsible for nearly 100% of all clinical malaria cases and children under five years and pregnant women are the populations at highest risk. Study design and details were previously described (31). Briefly, children were randomized to either receive three doses of GMZ2/alum or rabies vaccine on days 0, 28 and 56 and were passively followed in the ensuing months for febrile malaria episodes up to month 12 from the last vaccine dose. Any child reporting to the local health facility and/or to study team with fever or history of fever 48 hours prior to reporting at the health facility had peripheral blood taken for malaria parasitaemia determination by microscopy. Febrile malaria episode was defined as parasitaemia count of ≥ 5000 parasites/µl and fever or history of fever within the past 48 hours prior to reporting sick. Since age-dependent pyrogenic thresholds have not been determined in the present study, which is spanning multiple age groups and transmissions seasons, we have used a single parasite threshold throughout. Sera were collected at scheduled intervals between May, 2011 and February, 2012 and stored at -80°C until this analysis. To assess immune responses following the GMZ2/alum immunization and the risk of clinical malaria, baseline (Day 0) sera and sera collected one month (Day 84) after final vaccine dose were used.

### Blood Smear for Malaria Parasite Detection

Thin and thick blood films were prepared from a finger prick. The thin film was fixed with methanol for a few seconds. Both blood films were then stained with 10% Giemsa stain for 15 minutes for malaria parasite identification and quantification. The stained blood smears were rinsed with running tap water for about 10 seconds and allowed to air dry. Malaria parasites were counted (trophozoites) against 200 white blood cells (WBCs) on the thick film by two independent experienced microscopist using a light microscope under oil immersion at 100x magnification. Negative result was assigned after examining 200 high power fields of the thick film at x100 magnification. Parasite counts were converted to parasites density/μL of blood assuming 8000 WBCs/μL of blood. Malaria species identification was done using thin blood smears.

### Multiplex Luminex Assay for Antibody Quantification

IgG antibody levels were determined against a panel of 11 antigens (nMSP3-K1, MSPDBL2, GLURP-R2, MSP6, MSP3.3, MSP3.7, MSP2-3D7, SERA5, *Pf*38, *Pf*12 and MSP1-19) and GMZ2 in a multiplex assay as described elsewhere (32). Briefly, recombinant proteins were coupled to 1.25x10^7^ microspheres beads per bead region. 100µL of the beads mixture containing 1250 beads per bead region were added to a pre-wetted 96 well microtiter plate. Serum samples diluted at 1:1000 were added and incubated for 2 hours. A secondary antibody, phycoerythrin (PE) -labelled goat antihuman IgG (Jackson Immuno Research) was added at 1:3500 for the detection of IgG bound antibodies and incubated for 1 hour. For the quantification of IgG subclasses, mouse antihuman IgG1 or IgG3 diluted 1:5000 were added followed by a PE-labelled goat antimouse IgG diluted 1:200. Between steps, plates were washed 3 times each with assay buffer E (ABE: PBS [pH = 7.4], 0.1% bovine serum albumin [BSA], 0.02%Tween 20 and 0.05% sodium azide). Mean fluorescent intensity (MFI) was measured with Luminex 200 Bio-Plex analyser (Bio-Rad Laboratories, Inc.).

### Statistical Analysis

Data were analyzed using Stata version 15 (College Station, Texas) and GraphPad Prism version 8. Differences in geometric mean antibody levels were compared using a t-test after log transforming antibody data to base 10. Cox regression was used to estimate vaccine efficacy and to determine the association of antibody levels with incidence of clinical malaria, using a robust standard error to allow for repeated events in the same child. To compare effects by age group and time period, Wald tests were used to assess interactions. To standardise the antibody levels to the 12 antigens, levels were transformed to logarithms and the logged values, x, then transformed to z-scores $\frac{\left(x-\bar{x}\right)}{s}$ , where $\bar{x}$ is the mean and s the standard deviation of the logged values.

## Results

### Baseline Characteristics

The phase 2b efficacy trial of GMZ2/alum was conducted at 5 sites in East- West- and Central-Africa (31). The present analysis include participants from one of the sites, Banfora, a village with high malaria transmission in Burkina Faso where 590 children were randomized. Of these, 547 received all three doses of the vaccine (272 in the GMZ2 group and 275 in the rabies vaccine group). Samples and data were available from 453 children (82.82% of the ATP population), 223 in the GMZ2 group and 230 in the rabies vaccine group. The distribution of gender, age, and bed net use were similar in the two groups (**Table 1**).

**Table 1:** Demographic characteristics of participants by trial arm, stratified by age and gender.

Variable		Rabies group n = 223 (%)	GMZ2 group n = 230 (%)
Age category	12 - 35 months	120 (53.8)	104 (45.2)
	36 – 60 months	103 (46.2)	126 (54.8)
Gender	Female	104 (46.6)	114 (49.6)
	Male	119 (53.4)	116 (50.4)
Bed net use	No	32 (14.3)	38 (16.5)
	Yes	191 (85.7)	192 (83.5)

n = the number of children in a group; % = percentage out of the group.

### Febrile Malaria Episodes During Follow-Up

**Figure 1:**
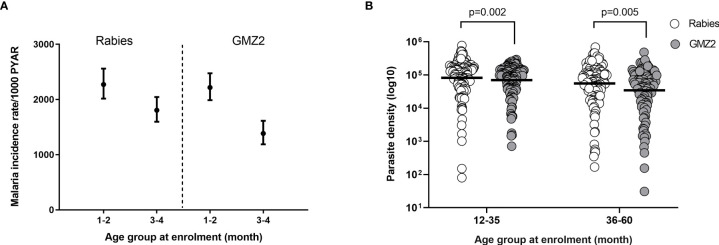
Clinical malaria incidence rate per randomization arm and parasite density by age group. **(A)** Incidence rate of febrile malaria by vaccine group per 1000-person year at risk (PYAR). **(B)** Geometric mean *P. falciparum* density by age group in both vaccine groups. P values were determined by t-test after log (base 10) transforming parasite density. Error bars represent geometric mean with 95% confidence intervals.

During the 12-months of follow-up (after dose 3 of vaccine was administered), 98.9% of the malaria episodes were *P. falciparum* mono-infections. The remaining episodes were mixed infections of *P. falciparum* and *P. ovale* or *P. malariae*. Children from the study cohort were stratified into younger (12-35 month) and older (36-60 month) age groups (based on age groups reported in the phase 2b trial) (31). The incidence of malaria episodes decreased per 1000 person years at risk with increasing age in both vaccine groups (**Figure 1A**) consistent with age-dependent acquisition of naturally acquired immunity (NAI) in the study population. We also plotted the geometric mean parasite density of each age group for all malaria cases (**Figure 1B**). The geometric mean parasite densities during febrile malaria cases decreased with increasing age. Importantly, geometric mean parasite densities were significantly (t-test, p ≤ 0.005) lower in the GMZ2 compared to the rabies groups in both age groups (**Figure 1B**).

### Vaccine Efficacy Is Age-Dependent in the Study Population

**Figure 2:**
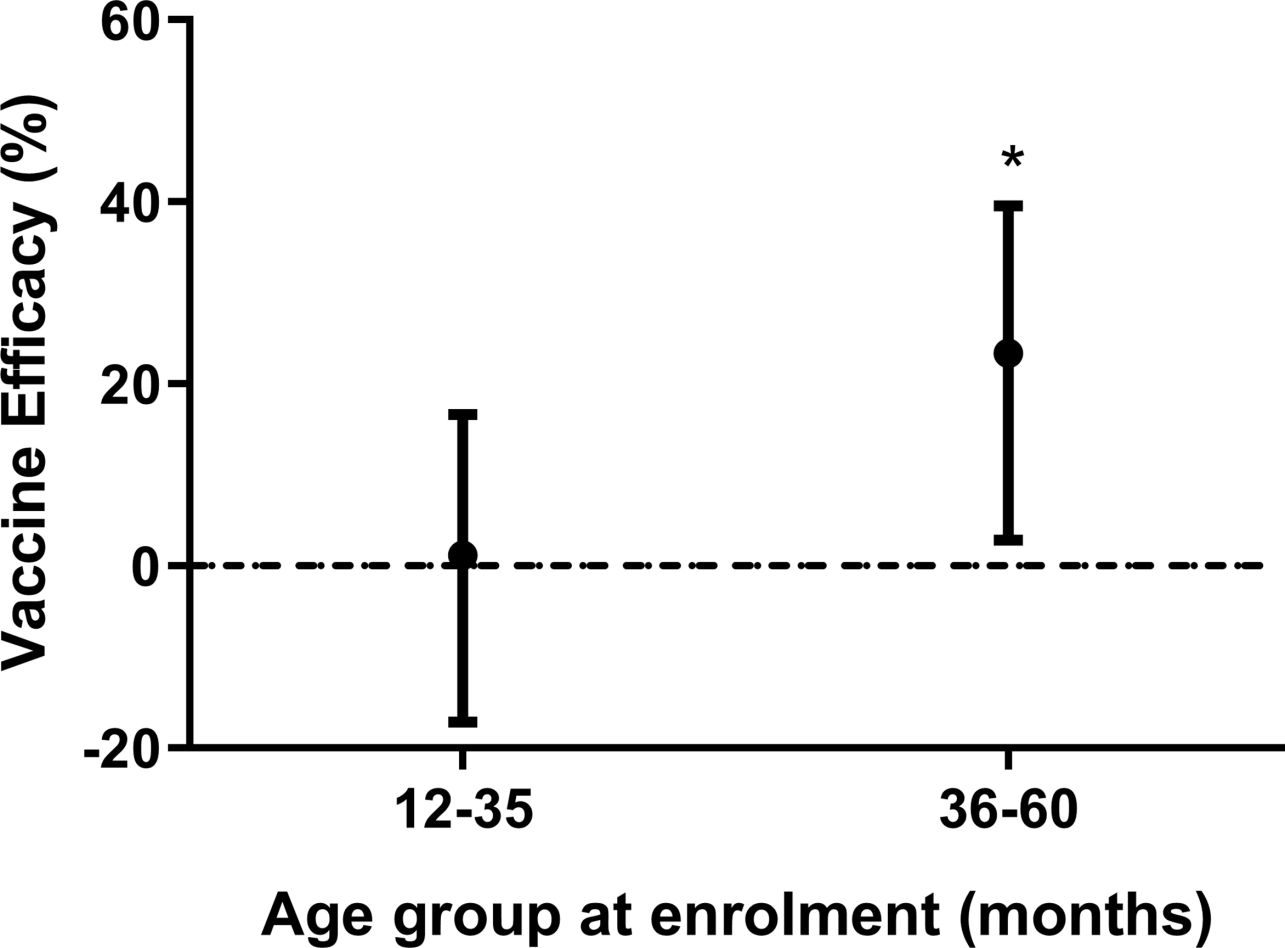
Vaccine efficacy stratified by age. Vaccine efficacy (VE) (using parasite density ≥ 5,000/uL and fever/history of fever) after 12 months of follow up stratified by age group. Error bars represent 95% confidence intervals. Cox regression model was used to calculate hazard ratios, 95% confidence intervals and p values for each age group. VE was defined as 100 × (1-HR), where HR is the hazard ratio from the Cox regression. The horizontal dashed line indicates (VE = 0). Asterisks represent P values (*P < 0.05).

We have previously observed a significant vaccine efficacy (VE) in the ATP analysis (31). In Banfora, we found that VE (using parasite density ≥ 5,000/uL and fever/history of fever) was higher in older children during the twelve months of follow-up (**Figure 2**). VE was 1% (95% confidence interval [CI] -17%, 17%) and 23% ([CI] 3%, 40%) in the younger and older children, respectively (**Figure 2**). To identify antibody specificities involved in VE, we investigated not only vaccine-induced antibodies but also naturally acquired antibodies against merozoite surface proteins not present in the vaccine because such antibodies may act in concert with GMZ2 antibodies.

### GMZ2 IgG Increase With Vaccination and Correlates With Decreased Parasitaemia

**Figure 3:**
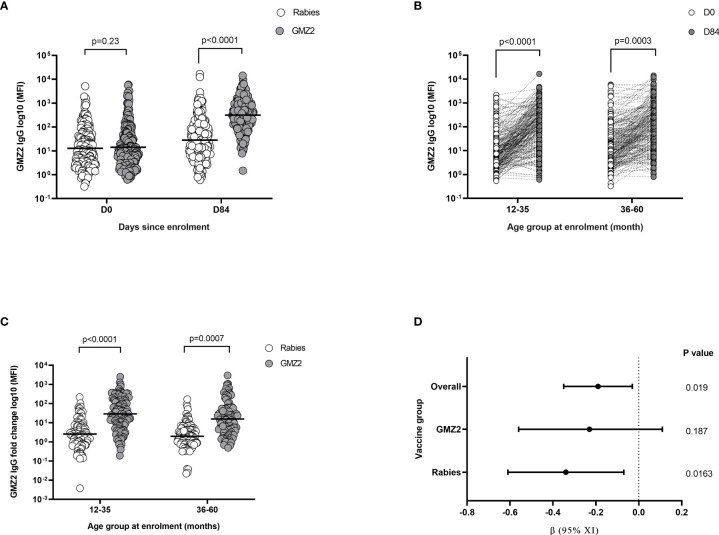
Antibody responses after GMZ2 vaccination and association with parasite density. **(A)** Mean fluorescent intensity (MFI) representing GMZ2 specific IgG levels compared between the two vaccine groups at day 0 (D0) and day 84 (D84). **(B)** Mean fluorescent intensity (MFI) representing GMZ2 specific IgG levels compared between day 0 (D0) and day 84 (D84) for the two age groups. **(C)** Fold increase (day 84 GMZ2-IgG/day 0 GMZ2-IgG) in GMZ2-IgG levels between day 0 and day 84 compared between the vaccine groups for each age group. All P values in panels **(A–C)** respectively were determined by t-test after log (base 10) transforming data. Horizontal lines represent geometric means. **(D)** Association between GMZ2 specific IgG levels and parasite density during febrile malaria in the overall study population, the GMZ2 vaccine group alone and the rabies vaccine group alone. Beta (β) coefficients, confidence intervals and p values were calculated using separate multiple linear regressing models adjusting for age. Antibody and parasite density data were both log (base 10) transformed prior to use in the models. The vertical dotted line indicates no association with between GMZ2 IgG and parasite density (β = 0).

GMZ2 IgG levels were first compared between vaccine groups at days 0 and 84 (one month after final vaccine dose), respectively. Levels of GMZ2 IgG were similar between the vaccine groups at day 0, however, at day 84, the GMZ2 group had significantly higher levels than the rabies group (t-test, p < 0.001) (**Figure 3A**). To assess whether antibody boosting depends on age, we compared GMZ2 antibody levels at days 0 and 84 for each age group. GMZ2 IgG levels were higher at day 84 compared to day 0 in both age groups (**Figure 3B**). The fold increase in GMZ2 specific IgG (i.e. day 84 GMZ2-IgG/day 0 GMZ2-IgG) was significantly higher in GMZ2 vaccinated children than those in the rabies vaccine group for each age group (**Figure 3C**). While the fold increase in the rabies group reflects natural exposure the increase in GMZ2 IgG levels in the GMZ2 vaccine group is a result of vaccination as well as natural exposure. Finally, we assessed the effect of a 10-fold increase in GMZ2 antibody level on parasite densities during febrile malaria in each vaccine group and in the overall cohort in separate multiple linear regression analysis adjusting for age of children. There was a significant decrease in parasitaemia associated with a 10-fold increase in GMZ2 specific antibodies in the overall study population and in the rabies group. However, although the same trend was observed in the GMZ2 group, the decrease in parasitaemia was not statistically significant [β = -0.23, 95%CI=(-0.56;0.11), p = 0.187], (**Figure 3D**). This suggests other non-GMZ2 IgG antibodies may have contributed to the decreased parasitaemia observed in the GMZ2 group (**Figure 1B**).

### GMZ2 IgG Was Associated With Reduced Incidence of Febrile Malaria

**Figure 4:**
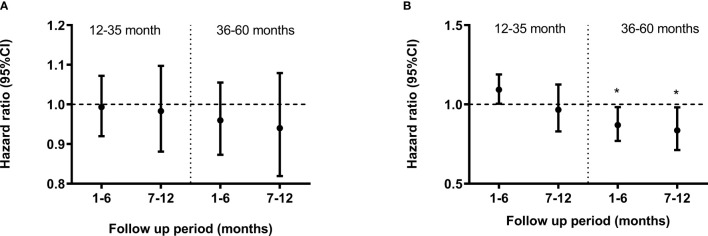
Association between GMZ2 antibody levels and clinical malaria stratified by age group. Cox regression model was used to calculate hazard ratios, 95% confidence intervals and p values for antibody levels in the rabies vaccine group **(A)** and GMZ2 vaccine group **(B)** for each age group. Error bars represent 95% confidence intervals. The horizontal dashed line indicates no association with protection (HR = 1). Asterisks represent P values (*P < 0.05). Malaria episodes were collected over 12 months of follow up.

The relationship between GMZ2 IgG levels on day 84, and the incidence of febrile malaria from that time point until 12 months post dose 3, was investigated separately in each vaccine group. The association differed by age group (interaction p-value 0.011). In the rabies group, there was no association between GMZ2 IgG levels and incidence of malaria in any of the age groups at any of the defined follow up periods (months 1-6 and 7-12, respectively) (**Figure 4A**). Similarly, in the GMZ2 group, there was no association between levels of GMZ2 IgG and malaria incidence in the younger children at any of the defined follow up periods. However, in the older children, GMZ2 IgG levels were significantly associated with reduced incidence of malaria during months 1-6 [hazard ratio (HR) = 0.87, 95%CI = (0.77, 0.98)] and 7-12 [HR = 0.84, 95%CI = (0.71, 0.98)] months of the follow-up period (**Figure 4B**).

### GMZ2 Vaccine Induced Antibodies Promote Opsonic Phagocytosis

**Figure 5:**
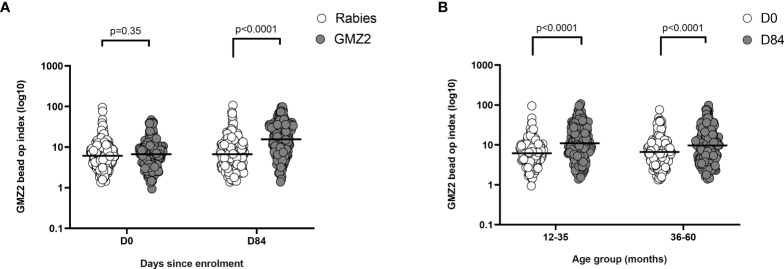
GMZ2 bead OP index in relation to age and vaccine group. **(A)** GMZ2 coated bead OP compared between the vaccine groups at day 0 (D0) and day 84 (D84). **(B)**. GMZ2 coated bead opsonic phagocytosis (OP) levels compared between day 0 (D0) and day 84 (D84) for the different age groups. P values were determined by t-test after log (base 10) transforming data. Horizontal lines represent geometric means.

Recently we developed a bead-based phagocytosis assay (BPA) to measure the functional activity of antibodies against distinct merozoite surface antigens (20). The GMZ2 vaccine antigen was immobilized on the surface of internally dyed microsphere beads and BPA activities of vaccine-induced antibodies were quantified in samples collected at days 0 and 84 from all study participants. A wide range of phagocytic activities were observed (**Figure 5**). At day 0, samples from both vaccine groups showed similar functional activities (**Figure 5A**). At day 84, there was a significant difference in BPA activity between the GMZ2 and rabies groups, demonstrating that GMZ2/alum elicit functional antibodies. The increase in functional activities was significant (t-test, p<0.0001) in both age groups (**Figure 5B**).

### Anti-Merozoite IgG Levels Increased in Both Vaccine Groups During Follow-Up

**Figure 6:**
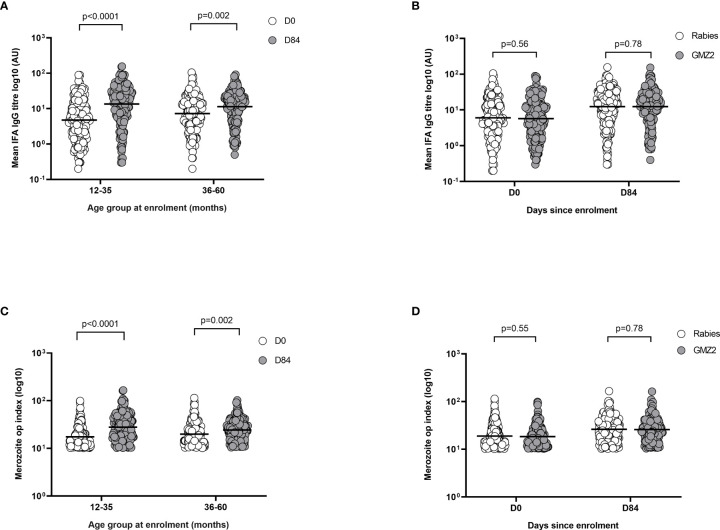
Merozoite IFA levels in relation to age and febrile malaria. **(A)** Merozoite IFA levels compared between day 0 (D0) and day 84 (D84) for the different age and vaccine groups. **(B)** Merozoite IFA levels compared between the vaccine groups at day 0 (D0) and day 84 (D84). **(C)** Merozoite opsonic phagocytosis (OP) index compared between day 0 (D0) and day 84 (D84) for the different age groups. **(D)** Merozoite opsonic phagocytosis (OP) index compared between the vaccine groups at day 0 (D0) and day 84 (D84). P values were determined by t-test after log (base 10) transforming data. Error bars represent geometric mean and 95% confidence intervals. IFA, Immunofluorescence assay; AU, Antibody unit.

First, we used a flow cytometry-based immunofluorescence assay (FC-IFA) to quantify anti-merozoite antibodies (33). In each age group, day 84 levels of merozoite IgG were higher than those at baseline (day 0) irrespective of the vaccine group (**Figure 6A**). When the study population was categorized according to vaccine group, there was no difference between merozoite IgG levels in children who received GMZ2/alum and those who received the rabies vaccine at either day 0 or day 84 (**Figure 6B**). In contrast, levels of merozoite IgG did increase between days 0 and 84 in either vaccine group suggesting the contribution of naturally acquired antibody boosting. Next, we used the merozoite opsonic phagocytosis (OP) assay to assess the functional activity of anti-parasite IgG at day 0 and day 84 (8, 24). Irrespective of the vaccine group, day 84 merozoite OP values were higher than day 0 values in both younger and older children (**Figure 6C**). However, there were no differences when merozoite OP values were compared between the vaccine groups on day 0 and day 84, respectively (**Figure 6D**).

Collectively, these findings are consistent with the notion that anti-merozoite immunity develops in the present study population irrespective of vaccine group.

### Dissecting Merozoite Specific IgG Responses Associated With Febrile Malaria in the Study Population

Having shown through the FC-IFA that natural exposure boosted anti-merozoite IgG responses in the study population, we next sought to delineate the potential specific merozoite antigens involved. Levels of merozoite specific antibodies were measured against a panel of 11 merozoite antigens not present in GMZ2 (**Supplementary Table S1**). There was a high variability in the IgG levels for the different antigens in the different vaccine groups. However, for each antigen, day 84 IgG levels appeared higher than day 0 IgG levels irrespective of the vaccine group suggesting a boosting through natural exposure (**Supplementary Table S1**). After transforming to logarithms (base=10) and calculating a z-score for the transformed variable, the association between incidence of malaria and day 84 antigen-specific antibody levels was assessed using Cox regression with a robust standard error to allow for repeated events within the same child and adjusted for age group and trial arm (GMZ2/alum or rabies vaccine) (**Table 2**). Antibodies against MSP3-K1 (HR = 0.87, 95% CI: 0.81 - 0.94 for a unit increase in z-score), MSPDBL2 (HR = 0.89, 95% CI: 0.83 - 0.97), GLURP-R2 (HR = 0.90, 95% CI: 0.84 - 0.97) and MSP3.7 (HR = 0.93, 95% CI: 0.87 – 1.00) were associated with reduced incidence of malaria in the study population. There was no evidence that the associations differed between the arms of the trial. Antibodies against all 11 antigens were then entered into the model to obtain estimates of independent association for each antibody adjusted for effects of the other antibodies. In the multivariate analysis, antibodies against only two antigens, MSP3-K1 (HR = 0.88, 95% CI: 0.80 – 0.97, p = 0.007) and GLURP-R2 (HR = 0.88, 95% CI: 0.80 – 0.98, p = 0.015), were independently associated with reduced incidence of malaria (**Table 2**). In the multivariate model, age remained strongly associated with malaria incidence indicating that the panel of immune responses measured only partially explained the reduction in incidence with age. A limitation is that we were not able to measure exposure to malaria, an important confounder, we may therefore have underestimated the strength of associations. Nonetheless, the data suggest that antibodies against MSP3-K1 and GLURP-R2 may have independently contributed to reducing malaria incidence in the study population during the clinical trial period and future GMZ2 designs could benefit from their incorporation.

**Table 2:** Association of non-GMZ2 antibodies with malaria incidence in the study population.

Variable	Hazard ratio ^a^ (95%CI)	P-value	AdjustedHazard ratio ^b^ (95%CI)	P-value
nMSP3-K1	0.87 (0.81,0.94)	<0.001	0.88 (0.80,0.97)	0.007
MSPDBL2	0.89 (0.83,0.97)	0.006	0.98 (0.88,1.09)	0.686
GLURP-R2	0.90 (0.84,0.97)	0.008	0.88 (0.80,0.98)	0.015
MSP6	0.92 (0.85,1.00)	0.054	1.01 (0.91,1.12)	0.901
MSP3.3	0.93 (0.86,1.00)	0.060	0.93 (0.86,1.01)	0.094
MSP3.7	0.93 (0.87,1.00)	0.048	0.96 (0.87,1.06)	0.375
SERA5	1.00 (0.93,1.07)	0.942	1.05 (0.96,1.15)	0.263
nMSP2-3D7	0.95 (0.88,1.02)	0.147	0.99 (0.90,1.10)	0.897
*Pf*38	1.00 (0.93,1.07)	0.935	1.01 (0.91,1.12)	0.867
*Pf*12	1.01 (0.94,1.09)	0.820	1.01 (0.91,1.11)	0.907
MSP1-19	1.04 (0.97,1.12)	0.283	1.10 (0.99,1.22)	0.092

^a^Association of each variable with malaria incidence, adjusted for age and trial arm.

^b^Independent association for each variable adjusted for effects of all the other variables.

95% confidence interval (95%CI). MSP, merozoite surface protein; MSPDBL2, merozoite surface protein duffy binding-like domain 2; GLURP-R2, glutamate rich protein region 2; SERA5, serine rich antigen 5; Pf, Plasmodium falciparum.

## Discussion

In summary, we showed VE in Banfora increased with increasing age of the children at enrolment, and older children (36-60 months) benefitted most from GMZ2/alum vaccination in the 12 months of follow-up. We further showed that naturally acquired antibodies to MSP3-K1 and GLURP-R2 measured at day 84 (one month after final vaccine dose), were associated with reduced malaria incidence in the study population during the trial period. Incorporation of these antigens in some form into future GMZ2 designs may help improve VE.

Considering all sites in the GMZ2 efficacy study, we observed that children in the GMZ2/alum group with high levels of GMZ2 IgG had a lower incidence of clinical malaria, after adjusting for age, compared with children with low levels (31). Here, we performed a series of association analyses in the GMZ2/alum vaccine group to further examine possible effects of GMZ2 IgG on protection against febrile malaria. Anti-GMZ2 antibody responses were investigated with respect to quantity and functional activity in the phagocytosis assay. Collectively, these assays demonstrated that GMZ2 vaccine elicited high levels of cytophilic IgG antibodies, which were capable of promoting phagocytosis of GMZ2-coated beads. Thus, supporting the notion that GMZ2 IgG may enhance merozoite-phagocytosis by blood leukocytes (24, 34). Contrary to expectations merozoite-phagocytosis was not stronger in the GMZ2 group compared to the rabies group suggesting that children in Banfora possesses relatively high levels of pre-existing anti-merozoite antibodies. We also, cannot rule out possibilities of antibodies against GMZ2 vaccine to mediate anti-malaria activity through other antibody-dependent mechanisms like Antibody Dependent Cellular Inhibition (ADCI) and inhibition of merozoite invasion,

We found that increasing anti-GMZ2 IgG levels were associated with reduced incidence of febrile malaria in older children 36-60 months of age during the first 12 months of follow-up. However, these analyses did not establish anti-GMZ2-IgG as the sole correlate of vaccine protection as they did not exclude potential confounders such as acquisition of antibodies to other blood stage antigens and age-dependent maturation of cell mediated immunity (35). Although IgG antibodies are thought to be the main effector molecule mediating protection against febrile malaria, cellular immune responses may also play a role through T-cell help for producing a robust antibody response or through multifunctional effector memory T cells producing IFN-γ, TNFα, and IL-2 (36). Whether GMZ2/alum enhance antigen-specific pluripotent lymphocytes remains to be investigated. Likewise, IgM antibodies may also play a role in malaria immunity. Recently, it was convincingly demonstrated that levels of specific IgM antibodies are associated with a reduced risk of clinical malaria in a longitudinal cohort study of children and that such antibodies may block merozoite invasion of red blood cells in a complement-dependent manner (37). Whether GMZ2-vaccine specific IgM antibodies play a similar role in the present cohort remains to be investigated.

We further observed that older children had lower parasite densities during febrile malaria attacks than the younger ones and that this difference was most pronounced in the GMZ2 vaccine group. This finding is consistent with observations that the parasite threshold at which fever is triggered depends on the age of the affected child. Older individuals were found to have a much lower pyrogenic threshold compared to younger ones (38, 39). When considering all study participants in Banfora, we further observed that increased levels of GMZ2 IgG were significantly associated with decreased parasitemias in these febrile attacks. This association was not observed in the GMZ2 group suggesting that these children have a lower pyrogenic threshold compared to children in the rabies vaccine group. It might be speculated that GMZ2 vaccination modulate the dynamics of parasitemia and the occurrence of fever. Pyrogenic cytokines Interlukin-1 IL1, IL6, and Tumor Necrosis Factor (TNF) are produced in response to malaria parasites (40). Of these, IL6, together with prostaglandin E2 (PGE2), is considered to be a major pyrogenic mediator of fever (reviewed in (41). Whether GMZ2 vaccination is affecting pyrogenic cytokine production and modulation of pyrogenic threshold triggered by malaria parasites through this inflammatory cytokines-neuronal body temperature regulatory axis mechanism remains to be determined.

It has previously been proposed that multiple anti-merozoite antibody specificities act in concert to provide protection against clinical malaria (17, 20) after a certain threshold has been reached (17). To determine whether several antibody specificities might also be involved in reducing clinical malaria incidence in Banfora, levels of distinct antibody specificities were assessed in the study population. We found, in a multivariate analysis involving eleven naturally acquired antibodies where the association of each antibody is adjusted for the effect of all the others, that levels of IgG against MSP3-K1 and GLURP-R2 were independently associated with reduced incidence of clinical malaria. Interestingly, both of these protein sequences are related to the GMZ2 constituent antigens, as they are derived from MSP3 and GLURP, respectively. The MSP3 antigen is a well-established target of naturally acquired immunity (42, 43) and analysis of sequences from most parasite isolates from malaria endemic populations show a distinct dimorphism belonging to either MSP3-3D7 or MSP3-K1 type alleles (44). In a Kenyan study, MSP3-K1 specific IgG was significantly associated with reduced risk of clinical malaria after adjusting for the effect of antibodies against other antigens such as AMA1 and MSP2 (45). Similarly, antibodies against GLURP-R2 have been associated with protection against malaria in several endemic populations including Burkina Faso, Ghana, and India (9, 14, 46). The finding that naturally acquired antibodies against variable regions of GLURP and MSP3 are associated with protection against febrile malaria in children from Banfora support the notion that antibodies against both conserved and variable domains are involved in protective mechanisms (16, 42, 47). While antibodies against the variable domains are thought to contribute to allele-specific immunity (42), antibodies against the conserved domains may provide protection against multiple parasite strains prevailing in the endemic population [reviewed in (48)]. Taken together, these findings suggest that future design of GMZ2 may benefit from the inclusion of variable epitopes from the MSP3 and GLURP antigens to improve efficacy. Whether such vaccine specific responses would be strain-specific remains to be investigated.

Other blood-stage malaria vaccines such as MSP-1 and AMA-1 intended to block or reduce the invasion of erythrocytes by malaria merozoites (4, 49, 50) have either shown no or little protection in Phase 2b efficacy studies (51–54). The main reason for these failures might be related to difficulties associated with the production of recombinant antigens with native conformations. However, polymorphisms observed for several of these malaria antigens in different parasite strains may also explain the lack of protective efficacy. Allele-specific vaccine efficacy has been reported in multiple trials of malaria vaccines, such as AMA1 (55), RTS,S (56) as well as those containing attenuated whole sporozoites (57). Considering the worldwide dynamics of *P. falciparum* parasites with different distributions among different regions and the finding that parasites may evolve over time possibly as a result of immune selection [reviewed in (58)], polymorphisms in key malaria antigens is considered a major obstacle to vaccine development. Although, the GMZ2 constituent antigens are relatively conserved (11, 59), it is possible that the limited VE might be due to some degree of strain-specific immunity. Overall, allelic-specific protection analysis of the GMZ2 trial may provide critical insights into putative strain-specific responses resulting in the development of more efficacious vaccine.

In conclusion, GMZ2/Alhydrogel VE was more pronounced in older children, and this may reflect a synergistic interaction between vaccine-induced and naturally acquired immune responses. Interestingly, additional epitopes from the variable regions of GLURP and MSP3 were identified as potential candidates for inclusion in future GMZ2 designs for improved efficacy. The study contributes important insights that could be useful in developing more efficacious blood-stage malaria vaccines that will benefit from a positive influence of naturally acquired immunity.

## Data Availability Statement

The original contributions presented in the study are included in the article/**Supplementary Material**. Further inquiries can be directed to the corresponding authors.

## Ethics Statement

The local Ethics Committees and regulatory authorities for Burkina Faso, Gabon, Ghana and Uganda reviewed and approved the clinical trial protocol before the start of the trial. Signed informed consent was obtained from parent/guardian of children before their inclusion in the study. The protocol was registered with the Pan African clinical trial registry with registration number ATMR2010060002033537.

## Author Contributions

SD, RT, and SKS performed the experiments. MT and SBS designed the clinical study. BA and MT designed the experiments and analysis. BA and MT wrote the manuscript. All authors reviewed the manuscript. All authors contributed to the article and approved the submitted version.

## Funding

This study was supported by grants from the European and Developing Countries Clinical Trials Partnership (grant IP.2007.31100.001), the German Federal Ministry of Education and Research (BMBF, grants 01KA0804 and 01KA1402) and Ministry of Foreign Affairs of Denmark (DFC file no.14-P01-GHA).

## Conflict of Interest

The authors declare that the research was conducted in the absence of any commercial or financial relationships that could be construed as a potential conflict of interest.

## Publisher’s Note

All claims expressed in this article are solely those of the authors and do not necessarily represent those of their affiliated organizations, or those of the publisher, the editors and the reviewers. Any product that may be evaluated in this article, or claim that may be made by its manufacturer, is not guaranteed or endorsed by the publisher.
