# Supplementary material for: GMZ2 Vaccine-Induced Antibody Responses, Naturally Acquired Immunity and the Incidence of Malaria in Burkinabe Children
Source: Front Immunol. 2022 Jun 2;13:899223. doi: 10.3389/fimmu.2022.899223 (PMC9200992; doi:10.3389/fimmu.2022.899223)
Supplement: Supplementary file 1 [file Table_1.docx]

Supplementary Table 1. Levels of IgG antibodies (median and interquartile range) against the vaccine antigen and antigens not present in the vaccine as determined through multiplex analysis. Antibody levels are expressed as mean fluorescence intensity (MFI) values.

|  | GMZ2 Group (n=230) | | Rabies Group (n=223) | |
| --- | --- | --- | --- | --- |
| Antigen^a^ | Day 0  Median (IQR) | Day 84  Median (IQR) | Day 0  Median (IQR) | Day 84  Median (IQR) |
|  | | | | |
| Vaccine antigen | | | | |
| GMZ2 | 10 (3-42) | 316 (121-936) | 9 (3-45) | 23 (7-90) |
|  | | | | |
| Periferally associated | | | | |
| nMSP3^K1^ | 185 (54-1096) | 481 (142-2110) | 189 (46-800) | 415 (147-1677) |
| MSPDBL2 | 165 (11-1960) | 520 (152-3250) | 109 (13-1978) | 257 (31-2419) |
| GLURP-R2 | 51 (17-408) | 209 (59-1536) | 61 (18-284) | 221 (55-1372) |
| MSP6 | 78 (29-380) | 133 (51-504) | 64 (28-260.69) | 154 (54-432) |
| MSP3.3 | 204 (81-651) | 398 (171-978) | 205 (85-732) | 299 (118-853) |
| MSP3.7 | 140 (34-867) | 447 (118-1561) | 109 (27-509) | 404 (147-1435) |
| SERA5 | 12 (3-56) | 31 (8-143) | 10 (3-45) | 30 (9-131) |
|  | | | | |
| Merozoite surface | | | | |
| MSP2^3D7^ | 427 (47-3445) | 2091 (253-8805) | 260 (66-1958) | 1605 (275-6043) |
| Pf38 | 43 (19-161) | 424 (144-1446) | 44 (16-158) | 139 (39-718) |
| Pf12 | 562 (291-1150) | 855 (464-2277) | 473 (263-1392) | 1012 (414-3007) |
| MSP1_19_ | 861 (188-3111) | 2117 (640-13889) | 71 (204-2316) | 2488 (744-11430) |

^a^ Recombinant proteins were derived from membrane anchored *P. falciparum* antigens and from antigens which are peripherally associated with merozoite surface.
